# Mercury, Lead, Cadmium, Arsenic, Chromium and Selenium in Feathers of Shorebirds during Migrating through Delaware Bay, New Jersey: Comparing the 1990s and 2011/2012

**DOI:** 10.3390/toxics3010063

**Published:** 2015-02-06

**Authors:** Joanna Burger, Nellie Tsipoura, Lawrence J. Niles, Michael Gochfeld, Amanda Dey, David Mizrahi

**Affiliations:** 1 Division of Life Sciences, Rutgers University, 604 Allison Road, Piscataway, NJ 08854-8082, USA; 2 Environmental and Occupational Health Sciences Institute (EOHSI), Piscataway, NJ 08854, USA; E-Mail: gochfeld@eohsi.rutgers.edu; 3 New Jersey Audubon, 11 Hardscrabble Rd, Bernardsville, NJ 07924, USA; E-Mails: nellie.tsipoura@njaudubon.org (N.T.); david.mizrahi@njaudubon.org (D.M.); 4 Conserve Wildlife, 109 Market Land, Greenwich, NJ 08323, USA; E-Mail: larry.niles@gmail.com; 5 Environmental and Occupational Medicine, Rutgers Robert Wood Johnson Medical School, Piscataway, NJ 08854, USA; 6 Endangered and Nongame Species Program, NJ Department of Environmental Protection, Trenton, NJ 08608, USA; E-Mail: amanda.dey@dep.state.nj.us

**Keywords:** mercury, selenium, molar ratios, birds, shorebirds, red knot, sanderling, semipalmated sandpiper, temporal patterns

## Abstract

Understanding temporal changes in contaminant levels in coastal environments requires comparing levels of contaminants from the same species from different time periods, particularly if species are declining. Several species of shorebirds migrating through Delaware Bay have declined from the 1980s to the present. To evaluate some contaminants as cause for the declines, we examine levels of mercury, lead, cadmium, arsenic, chromium and selenium in feathers of red knot (*Calidris canutus*, *N* = 46 individuals), semipalmated sandpiper (*Calidris pusilla*, *N* = 70) and sanderling (*Calidris alba*, *N* = 32) migrating through Delaware Bay, New Jersey, USA, from 1991 to 1992 (*N* = 40), 1995 (*N* = 28), and 2011–2012 (*N* = 80) to determine if levels have changed. We found: (1) arsenic, chromium, and lead increased in red knot and decreased in semipalmated sandpiper; (2) cadmium decreased in semipalmated sandpipers; (3) mercury decreased in red knot and sanderlings; (4) selenium decreased in red knot and increased in semipalmated sandpipers. In 2011/2012 there were significant interspecific differences for arsenic, mercury and selenium. Except for selenium, the element levels were well below levels reported for feathers of other species. The levels in feathers in red knots, sanderling, and semipalmated sandpipers from Delaware Bay in 2011/2012 were well below levels in feathers that are associated with effect levels, except for selenium. Selenium levels ranged from 3.0 µg·g^−1^ dry weight to 5.8 µg·g^−1^ (semipalmated sandpiper), within the range known to cause adverse effects, suggesting the need for further examination of selenium levels in birds. The levels of all elements were well below those reported for other marine species, except for selenium, which was near levels suggesting possible toxic effects.

## 1. Introduction

With global change and increasing levels of industrial, commercial and agricultural contaminants it is important to track temporal trends and compare species, particularly species of conservation concern, or those living or migrating through unique or sensitive habitats. Since the 1980s, Delaware Bay has been recognized as a migratory stopover of “hemispheric importance” in the Western Hemisphere Shorebird Reserve Network. In the 1990s, as many as 270,000 shorebirds were recorded in a single count, and estimates were made of more than 1 million shorebirds using the Bay during spring migration [1]. This represents the largest concentration of northbound shorebirds on the east coast of the United States.

During the 2–3 week stop-over at Delaware Bay, shorebirds eat intensely and store fat to refuel for the last 4000+ mile leg of their journey to the Canadian Arctic and subarctic breeding grounds. Their migration through Delaware Bay in May coincides with the spawning of the horseshoe crabs (*Limulus polyphemus*) [2], whose eggs they consume in large quantities as they are easy to find, digest, and metabolize [3]. To successfully make the long migration and arrive in condition to lay eggs immediately upon arrival at the breeding grounds, the shorebirds need to nearly double their weight during May [4].

However, horseshoe crabs, especially egg-laden females, are harvested as bait for eel and conch fisheries. As harvest levels increased in the early to mid-90s, horseshoe crab populations declined significantly along with the abundance and availability of their eggs to shorebirds [5]. Horseshoe crabs deposit their eggs in nests below the sand surface during high tides. These eggs are unavailable to the foraging shorebirds and gulls, until subsequently arriving females dig up nests of other females, releasing the eggs to the surface. The shorebirds are thus feeding on excess eggs, and in the 1990s, the tide line along the beach was pale green with masses of eggs [6], while in the early 2000s excess eggs were scarce.

The number of red knots *Calidris canutus*, semipalmated sandpipers *Calidris pusilla* and other shorebirds using Delaware Bay declined over the past fifteen years [5,7]. While there is strong evidence that shorebird declines are related to the decreased availability of horseshoe crab eggs [2,8,9], shorebird populations have experienced similar declines throughout their range [10,11]. Therefore, it is essential to explore other constraints on shorebird populations including habitat degradation, human disturbance, continued illegal hunting in the wintering grounds, and other anthropogenic factors, such as exposure to contaminants [12,13,14,15,16,17].

In this paper we examine levels of mercury, lead, cadmium, arsenic, chromium and selenium in feathers of red knot, semipalmated sandpiper and sanderling (*Calidris alba*) collected from Delaware Bay from 1991 to 1992 and 1995 compared with 2011 and 2012. No element analyses were conducted between these time periods, despite the significant shorebird declines. We were particularly interested in: (1) whether there were interspecific differences; (2) whether levels had changed from the 1990s to the present; and (3) whether the levels posed a risk based on known effects levels. We examined levels of mercury, cadmium, and lead because they are the major contaminants of concern in marine environments [18,19,20], arsenic, a concern for wildlife in marine and estuarine ecosystems [21], and chromium because it has posed a major environmental contamination problem from former industrial processes in northern New Jersey [22]. Some contaminants, such as methylmercury, were of special interest because they biomagnify up the food chain [23,24]. Selenium is both a highly toxic elementiand an essential trace element, which bears a complex relationship with mercury, hence our attention to this element [25].

## 2. Methods

### 2.1. Collecting Methods

**Figure 1:**
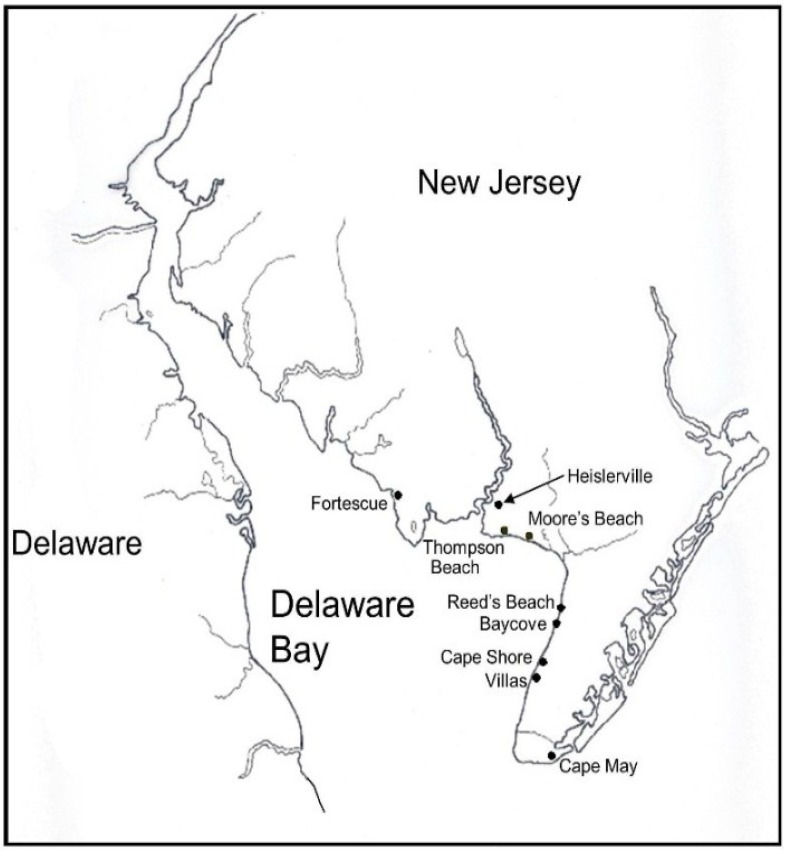
Map showing locations where feathers were collected from shorebirds migrating through Delaware Bay, New Jersey, USA, in the spring.

Our overall protocol, under appropriate state and federal permits was to collect feathers from adult shorebirds netted during their migratory stopover on Delaware Bay beaches in New Jersey (Cape May and Cumberland Counties). In 1990–1991 and 1995 [26], samples were collected from Moore’s Beach and Reed’s Beach. In 2011 and 2012 samples were collected from Reed’s Beach and the neighboring Kimble’s Beach, and from Heislerville and Fortescue, which are located adjacent to Moore’s Beach (Figure 1). All three species move back and forth, up and down the coast on a frequent basis among these sites which span about 20 km of beach, so we did not expect any differences due to the locality of the trapping.

Since the 1990s, over 3000 shorebirds have been captured yearly as part of an international effort to understand the biology, ecology, and migratory behavior of shorebirds on Delaware Bay. The primary team included scientists from New Jersey Audubon, New Jersey Department of Environmental Protection (Endangered and Nongame Species Program), and Conserve Wildlife Foundation. Birds were captured by rocket netting, whoosh netting, and mist netting. Size and weight measurements were recorded, and birds were marked with uniquely coded leg flags and leg bands. A small “pinch” of breast feathers (about 20–30) were collected from each bird, and placed in clear envelopes (in light-inhibiting boxes) for analysis at the Environmental and Occupational Health Sciences Institute of Rutgers University. Usually 10–15 feathers were required for analysis, so for small samples, all feathers were used. All methods were approved by the Rutgers University IRB (92-036), and conform to guidelines provided by the Ornithological Council [27]. These guidelines have been formulated with consideration of animal welfare and research needs. Due to changes in the detection levels and quantification of instrumentation in the 1990s compared to 2012, we re-analyzed the archived feather samples from the 1990s along with the 2011/2012 samples, although we did not have all the samples from the earlier time period (thus some differences were expected).

### 2.2. Chemical Analysis

Feathers were kept in envelopes at room temperature until they were transferred to the Elemental Analysis Laboratory of the Environmental and Occupational Health Sciences Institute at Rutgers University. Sample preparation, digestion, and analytic details have been published previously [28]. Feathers were washed three times with acetone, alternating with deionized water, and air dried. A 0.05 to 0.1 gram sample was digested in 4 mL 70% Trace Metal grade nitric acid (Fisher Scientific, Pittsburgh, PA, USA) and 2 mL deionized water in a microwave (CEM MDS 2000, Waltham, MA, USA). Total mercury was analyzed by cold vapor atomic absorption spectrophotometry using a Perkin-Elmer FIMS-100 mercury analyzer, Waltham, MA, USA). In feathers about 95% of the total mercury is methylmercury [29]. Other elements were analyzed using a Perkin-Elmer 5100 graphite furnace (flameless) atomic absorption spectrometer with Zeeman correction. Waltham, MA, USA. Instrument detection limits are 0.02 ng·g^−1^ for arsenic and cadmium, 0.08 ng·g^−1^ for chromium, 0.15 ng·g^−1^ for lead, 0.2 ng·g^−1^ for mercury and 0.7 ng·g^−1^ for selenium. All specimens were analyzed in batches with known standards, blanks, and spiked specimens. We also analyzed reference materials (trace elements in natural water, certified from the National Institute of Standards and Technology [NIST]). DORM-2 certified dogfish muscle, provided by NIST, was used as a reference material for mercury. Acceptable results for the reference materials were between 90% and 110%. Recoveries ranged from 88% to 102%. All concentrations are expressed in µg·g^−1^ (ppm), dry weight.

### 2.3. Data Analysis

We used non-parametric procedures (Kruskal Wallis non-parametric analysis of variance, PROC NPAR1WAY) [30], to determine species and temporal differences in element levels. We used these non-parametric tests because they are more conservative and are best suited for small datasets.

## 3. Results

There were significant temporal differences between samples from the 1990s and 2011–2012 for some elements, for some species (Table 1). There were significant increases in levels of: (1) arsenic, chromium and lead in red knot; and (2) selenium in semipalmated sandpiper. There were significant decreases in levels of: (1) arsenic, cadmium, and chromium in semipalmated sandpipers; (2) mercury in sanderling and red knot; (3) cadmium in semipalmated sandpipers, and selenium in red knot. Thus, the temporal patterns differed, but more element levels increased in feathers of red knot (arsenic, chromium, lead), more decreased in semipalmated sandpipers (arsenic, cadmium, chromium, and lead, and in sanderlings there were not significant differences (except declines in mercury). Arsenic, chromium, and lead showed opposite trends in knot (increase) and semipalmated sandpipers (decrease) while selenium showed the opposite trend in the same species (decrease in knot and increase in the sandpipers).

**Figure 2:**
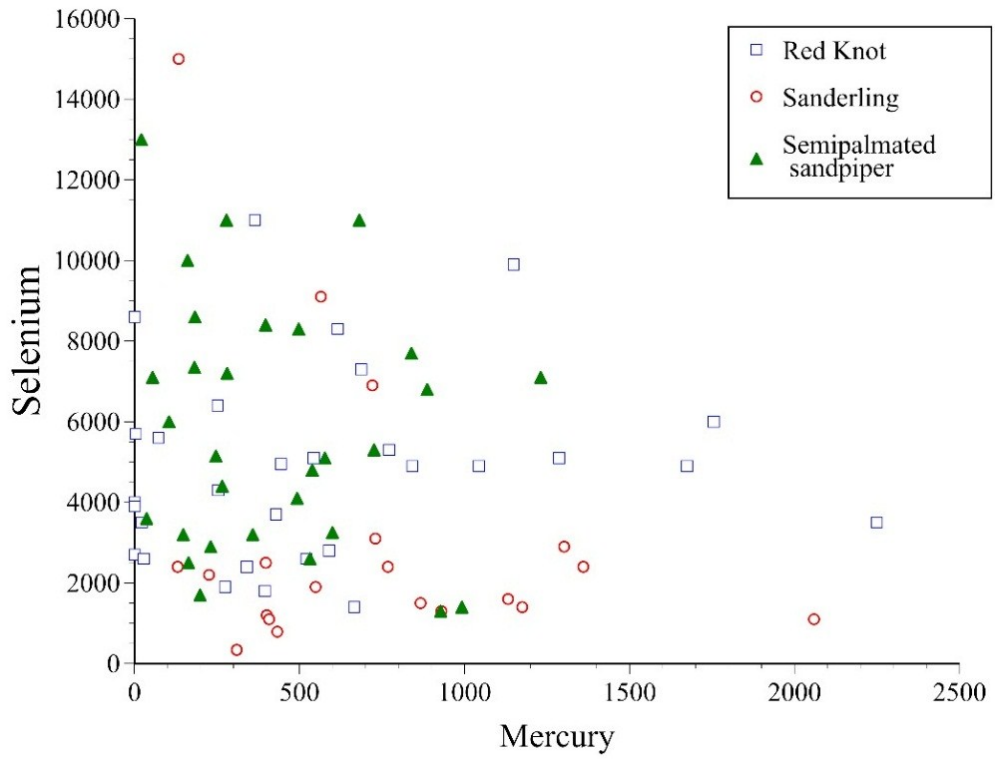
Mercury-selenium relationship in feathers of the three shorebirds.

Species differences in the 1991/1992 and the 2011/2012 periods were also examined (Table 1). Significant interspecific differences in levels in feathers in 1991/1992 occurred for all elements, with semipalmated sandpipers having the highest levels of all elements except mercury (sanderling was highest) and selenium (knot was highest). Significant differences in 2011–2012 occurred for arsenic, mercury and selenium. Semipalmated sandpipers had the highest levels of arsenic and selenium, and sanderling had the highest levels of mercury in feathers.

For many birds, mercury is the major element of concern, and selenium is thought to ameliorate the effects of mercury toxicity [31]. Although levels in feathers only represent circulating levels at the time of their formation, they are indicative of internal levels at that time. Therefore, we examined the relationship between mercury and selenium levels in feathers, and found that there was no significant correlation (*P* greater than 0.1) for each of the three species (Figure 2).

**Table 1:** Temporal patterns in element levels (ng·g^−1^, dry weight) in feathers of Red Knot, Sanderling, and Semipalmated Sandpipers from Delaware Bay, NJ, USA.

		1991–1992			1995			2011–2012			Temporal Kruskal-Wallis Chi-Square (*p*)
		Analyzed in 2013			Analyzed in 2013						
		Mean ± STD Error			Mean ± STD Error			Mean ± STD Error			
Red Knot	*n*		16						30		
	Arsenic	209	±	22				446	±	42	15.4 (<0.0001)
	Cadmium	19	±	4				17	±	2	NS
	Chromium	291	±	42				578	±	83	7.6 (0.006)
	Lead	139	±	13				484	±	67	24.3 (<0.0001)
	Mercury	791	±	77				576	±	105	5.0 (0.03)
	Selenium	7550	±	536				4835	±	432	13.0 (0.0003)
Sanderling	*n*		12						20		
	Arsenic	238	±	39				311	±	64	NS
	Cadmium	14	±	3				11	±	3	NS
	Chromium	764	±	260				463	±	62	NS
	Lead	268	±	52				367	±	52	NS
	Mercury	2007	±	352				730	±	109	8.5 (0.004)
	Selenium	2063	±	266				3057	±	781	NS
Semipalmated Sandpiper	*n*		12			28			30		
	Arsenic	1140	±	217	563	±	52	842	±	101	7.7 (0.02)
	Cadmium	48	±	9	25	±	7	14	±	3	18.1 (0.0001)
	Chromium	1149	±	294	450	±	66	523	±	64	11.0 (0.004)
	Lead	849	±	165	553	±	70	411	±	46	9.2 (0.01)
	Mercury	420	±	82	509	±	31	428	±	58	NS
	Selenium	3462	±	604	4165	±	434	5802	±	562	7.0 (0.03)
Species Comparisons Chi-Square (p)											
	Arsenic	15.0 (0.0005)						25.4 (<0.0001)			
	Cadmium	15.3 (0.0005)						2.9 (NS)			
	Chromium	12.1 (0.002)						0.7 (NS)			
	Lead	21.1 (<0.0001)						0.8 (NS)			
	Mercury	15.3 (0.0005)						5.1 (NS)			
	Selenium	24.8 (<0.0001)						17.6 (0.0001)			

## 4. Discussion

### 4.1. Temporal Patterns

In the present study, we found the following temporal patterns: (1) arsenic, chromium, lead, and selenium increased in one species, declined in another, and did not differ in another; (2) cadmium stayed the same in two species and declined in the third; and (3) mercury declined in two species and remained the same in the third. The results show concordant temporal pattern among species. There are no comparable studies that examined temporal patterns in elements in shorebirds from Delaware Bay or elsewhere.

Many chemicals, such as mercury, undergo atmospheric transport all over the world, including relatively isolated lakes and oceanic environments [32]. Atmospheric deposition of mercury is problematic because even developed industrialized nations do a poor job of regulating emissions [33], which suggests that mercury might not be declining. In the present study, there was no clear pattern in mercury levels in feathers of shorebirds, and Burger [28] found no decline in feathers of young egrets from nearby Barnegat Bay.

We expected declines in lead and cadmium because levels have declined in the environment generally from regulations for cadmium in batteries, and the removal of lead from paint and gasoline [34]. Declines in cadmium and lead occurred at the same time, although declines in cadmium were not as great as lead. Lead and cadmium from batteries and gasoline entered entering aquatic environments and were accumulated by invertebrates and fish, which were then consumed by birds (which then deposited them in feathers) [35]. In the present study, cadmium decreased or remained the same in the three shorebirds examined, but lead showed no clear pattern. Significant declines have been reported in levels of lead and cadmium in feathers of young egrets [28] and in eggs of common terns (*Sterna hirundo*) from Barnegat Bay [20].

There are few data on temporal patterns of the other elements in feathers for other birds, although Burger [28] found no significant declines for arsenic and chromium. In the present study, there were also no consistent patterns for these two elements, as well as for selenium.

### 4.2. Interspecific Comparisons

Since all three species forage on invertebrates, and appear to be at the same trophic level, we did not expect consistent differences in element levels, or great differences among the species in element levels. In 1991/1992, semipalmated sandpipers had the highest levels of all elements except mercury and selenium. In 2011/2012, however, there were significant interspecific differences for arsenic, mercury and selenium, but semipalmated sandpipers had the highest levels of arsenic and selenium. While levels of mercury declined in sanderling, they had significantly higher mercury levels than the other two species in both time periods. The sources of these differences are unclear, although they may relate to differences in foraging locations or prey on the wintering grounds, as some breast feathers are generally molted prior to migration (although this varies somewhat with species). Even on Delaware Bay the birds feed on different prey and in different locations some of the time. Sanderlings spend time foraging on the ocean beaches, semipalmated sandpipers sometimes forage on the salt marshes, and red knots mainly forage on the bay mudflats and beaches. Thus the differences in metal levels among species may indicate slightly different trophic levels, as has been found for shorebirds using mudflats along coastal France [36,37]. In the same species, trophic level could vary over two trophic levels indicating trophic plasticity [36,37].

The levels of all elements in feathers of the shorebirds examined were either well below those reported from several feather studies summarized in Burger [35], or they were similar (selenium). Mean selenium levels in feathers of marine birds, for example, averaged 6.0 µg·g^−1^, compared to 3.0 µg·g^−1^ in sanderling, 4.8 µg·g^−1^ in red knot, and 5.8 µg·g^−1^ in semipalmated sandpiper in the present study. Selenium levels in the shorebirds studied in this study were thus similar to those of marine birds, many of which spent most of their life at sea eating invertebrates and fish.

Metal levels in feathers are available for dunlin (*Calidris alpina*), and redshank (*Tringa totanus*) [36], and red knots and black-tailed godwits (*Limosa limosa*) [37]. The feathers of red knots collected in 2011–2012 from Delaware Bay were either lower than (arsenic, cadmium, lead, mercury and selenium) or similar (chromium) to those collected in France [36,37]. Similarly, the levels in feathers of sanderling and semipalmated sandpiper were lower for all metals [35].

### 4.3. Effects Levels

Contaminant exposure can have negative effects on reproduction, egg hatchability, hatchling survivorship and neurobehavioral development [38,39,40,41,42,43,44]. The elements of primary concern in marine environments are mercury, cadmium and lead [18] because they are non-essential, common, and highly toxic [44,45,46,47,48,49].

In contrast to most other metals, there has been considerable work on the effects of mercury in laboratory and field studies [43,46]. Mercury levels in feathers that are associated with adverse reproductive effects in birds are 5 µg·g^−1^ [42,44], although Jackson *et al.* [50] demonstrated effects at feather levels below 5 µg·g^−1^ (2.4 µg·g^−1^) in wrens (*Thryothorus ludovicianus*). Concentrations of 15 µg·g^−1^ mercury are required for adverse effects in some predatory birds [44]. The mean levels in the feathers of shorebirds in this study in 2011/2012 were 0.4 to 0.6 µg·g^−1^, which suggests that mercury is not posing a problem for these species.

Adverse effects in birds occur at lead levels of 4 µg·g^−1^ in feathers [35], although marine species can often tolerate higher levels [42]. In the present study, mean lead levels in 2011/2012 averaged 0.4 µg·g^−1^ to 0.5 µg·g^−1^, which was an order of magnitude below known effects levels.

Cadmium can cause adverse behavioral effects at lower concentrations than lead and mercury [44,49], including slow growth rates [19]. Feather levels known to cause adverse effects in the birds themselves range from 0.1 µg·g^−1^ (shearwaters) to 2 µg·g^−1^ (terns, Burger [35]). In this study, the mean cadmium levels in feathers of shorebirds in 2011/2012 averaged 0.01 µg·g^−1^, well below any effects levels.

Levels of selenium in feathers known to be associated with toxic effects, range from 1.8 µg·g^−1^ (sublethal) to 26 µg·g^−1^ (lethality), depending upon species [25,35,51]. The average level of selenium in feathers of shorebirds from Delaware Bay in 2011/2012 ranged from 3.0 µg·g^−1^ to 5.8 µg·g^−1^, which suggests some potential for adverse effects. Levels of effects for marine birds or shorebirds, however, have not been examined in the laboratory, so interpretation is difficult. High levels of selenium have been reported in shorebird tissues, including feathers at other sites [39,52]. The interaction of selenium and mercury, both known to ameliorate the toxicity of the other, requires more toxicological studies in a range of species [25,51]. Selenium and mercury levels showed no consistent patterns in feathers in the shorebirds we examined from Delaware Bay (Figure 2). This suggests that there may be no consistent relationship in blood, which in turn may suggest no ability to predict if toxicity is ameliorated, but this requires further examination.

There are few controlled laboratory studies for other elements, such as arsenic and chromium, and those that do related adverse effects to liver or kidney levels, rather than feathers, making it difficult to interpret the significance of the levels found in feathers of shorebirds.

## 5. Conclusions

Overall, where there were significant differences, levels of six elements in three species declined in seven cases out of 18 comparisons, and increased in four cases (there were no significant differences in seven cases). Thus, element levels generally declined or remained stable. The levels of all elements (except selenium) were well below those reported for feathers of other species (especially marine birds). Although selenium is an essential element and is believed to confer some protection against mercury toxicity, the levels in the present study approached toxic levels. Otherwise, the levels in feathers in red knots, sanderling, and semipalmated sandpipers from Delaware Bay in 2011/2012 were well below levels in feathers that are associated with effects.

## References

[1] Clark K., Niles L., Burger J. (1993). Abundance and distribution of shorebirds migrating on Delaware Bay, 1986–1992. Condor.

[2] Niles L.J., Sitters H.P., Dey A.D., Arce N., Atkinson P.W., Baker A.J., Bennett K.A., Buchanan J., Carmona R., Harrington B.A. (2008). Status of the Red Knot, *Calidris canutus rufa*, in the Western Hemisphere. Stud. Avian Biol..

[3] Tsipoura N., Burger J. (1999). Shorebird diet during spring migration stopover on Delaware Bay. Condor.

[4] Morrison R.I.G., Davidson N.C., Wilson J.R. (2007). Survival of the fattest: Body stores on migration and survival in red knots *Calidris canutus islandica*. J. Avian Biol..

[5] Niles L.J., Bart J., Sitters H.P., Dey A.D., Clark K.E., Atkinson P.W., Baker A.J., Bennett K.A., Kalasz K.S., Clark J. (2009). Effects of horseshoe crab harvest in Delaware Bay on Red Knots: Are harvest restrictions working?. BioScience.

[6] Burger J. (1996). A Naturalist along the Jersey Shore.

[7] Mizrahi D., Peters K., Tanacredi J.T., Botton M.L., Smith D.R. (2009). Relationships between Sandpipers and Horse Shoe Crabs in Delaware Bay: A synthesis. Biology and Conservation of Horseshoe Crabs.

[8] Kraemer G., Michels S., Tanacredi J.T., Botton M.L., Smith D.R. (2009). History of Horseshoe Crab Harvest in Delaware Bay. Biology and Conservation of Horseshoe Crabs.

[9] McGowan C.P., Hines J.E., Nichols J.D., Lyons J.E., Smith D.R., Kalasz K.S., Niles L.J., Dey A.D., Clark N.A., Atkinson P.W. (2011). Demographic consequences of migratory stopover: Linking red knot survival to horseshoe crab spawning abundance. Ecosphere.

[10] Bart J., Brown B., Harrington B.A., Morrison R.I.G. (2007). Survey trends of North American shorebirds: Population declines or shifting distributions?. J. Avian Biol..

[11] Andres B.A., Smith P.A., Morrison R.G., Gratto-Trevor C.L., Brown S.C., Friis C.A. (2013). Population estimates of North American shorebirds, 2012. Int. Wader Stud. Group.

[12] Burger J., Clark K.L., Niles L. (1997). Importance of beach, mudflat and marsh for migrant shorebirds on Delaware Bay. Biol. Conserv..

[13] Burger J., Jeitner C., Clark K.L., Niles L. (2004). The effect of human activities on migrant shorebirds: Successful adaptive management. Environ. Conserv..

[14] Burger J., Carlucci C., Jeitner C., Niles L. (2007). Habitat choice, disturbance, and management of foraging shorebirds and gulls at a migratory stopover. J. Coast. Res..

[15] Galbraith H., Jones R., Park R., Clough J., Herod-Julius S., Harrington B., Page G. (2002). Global climate change and sea level rise: Potential losses of intertidal habitat for shorebirds. Waterbirds.

[16] Goss-Custard J.D., Triplet P., Sueur F., West A.D. (2005). Critical thresholds of disturbance by people and raptors in foraging wading birds. Biol. Conserv..

[17] Hargreaves A.L., Douglas P., Whiteside R., Gilchrist G. (2011). Concentrations of 17 elements, including mercury, in the tissues, food and abiotic environment of Arctic shorebirds. Sci. Total Environ..

[18] Fowler S.W. (1990). Critical review of selected heavy metal and chlorinated hydrocarbon concentrations in the marine environment. Mar. Environ. Res..

[19] Spahn S.A., Sherry T.W. (1999). Cadmium and lead in exposure associated with reduced growth rates, poorer fledging success of Little Blue heron chicks (*Egretta caerulea*) in South Louisiana wetlands. Arch. Environ. Contam. Toxicol..

[20] Burger J., Gochfeld M. (2004). Metal levels in eggs of common terns (*Sterna hirundo*) in New Jersey: Temporal trends from 1971 to 2002. Environ. Res..

[21] Neff J.M. (1997). Ecotoxicology of arsenic in the marine environment. Environ. Toxicol. Chem..

[22] Burke T., Fagliano J., Goldoft M., Hazen R.E., Iglewicz R., McKee T. (1991). Chromite ore processing residue in Hudson County, New Jersey. Environ. Health Perspect..

[23] Burger J. (2002). Food chain differences affect heavy metals in bird eggs in Barnegat Bay, New Jersey. Environ. Res..

[24] Weis J.S. (2005). Diet and food web support of the white perch, *Morone americana* in the Hackensack Meadowlands. Environ. Biol. Fishes.

[25] Eisler R. (2000). Handbook of Chemical Risk Assessment: Health Hazards to Humans, Plants and Animals.

[26] Burger J., Seyboldt S., Morganstein N., Clark K. (1993). Heavy metals and selenium in feathers of three shorebird species from Delaware Bay. Environ. Monit. Assess..

[27] Ornithological Council Guidelines to the Use of Wild Birds in Research. http://www.nmnh.si.edu/BIRDNET/guide/guidelines.html.

[28] Burger J. (2013). Temporal trends (1989–2011) in levels of mercury and other heavy metals in feathers of fledgling great egrets nesting in Barnegat Bay, New Jersey. Environ. Res..

[29] Wolfe M., Norman D. (1998). Effects of waterborne mercury on terrestrial wildlife at clear lake: Evaluation and testing of a predictive model. Environ. Toxicol. Chem..

[30] SAS (2005). Statistical Analysis System.

[31] Ralston N.V.C., Raymond L.J. (2010). Dietary selenium’s protective effects against methylmercury toxicity. Toxicology.

[32] Hammerschmidt C.R., Fitzgerald W.F., Lamborg C.H., Balcom P.H., Tseng C.M. (2006). Biogeochemical cycling of methylmercury in lakes and tundra watersheds of Arctic Alaska. Environ. Sci. Technol..

[33] Evers D.C., Burgess N.M., Champous L., Hoskins B., Major A., Goodale W.M., Taylor R.J., Poppenga R., Daigle T. (2005). Patterns and interpretation of mercury exposure in freshwater avian communities in northeastern North America. Ecotoxicology.

[34] Agency for Toxic Substances and Disease Registry (ATSDR) (1997). Toxicological Profile for Lead. Agency for Toxic Substances and Disease Registry.

[35] Burger J. (1993). Metals in avian feathers: Bioindicators of environmental pollution. Rev. Environ. Toxicol..

[36] Lucia M., Bocher P., Chambosse M., Delaporte P., Bustamante P. (2014). Trace element accumulation in relation to trophic niches of shorebirds using intertidal mudflats. J. Sea Res..

[37] Lucia M., Bocher P., Cosson R.P., Churlaud C., Bustamante P. (2012). Evidence of species-specific detoxification processes for trace elements in shorebirds. Ecotoxicology.

[38] Heinz G.H. (1974). Effects of low dietary levels of methyl mercury on mallard reproduction. Bull. Environ. Contam. Toxicol..

[39] Ohlendorf H.M., Hothem R.L., Welsh D. (1989). Nest success, cause-specific nest failure, and hatchability of aquatic birds at selenium-contaminated Kesterson Reservoir and a reference site. Condor.

[40] Wolfe M., Schwarzbach S., Sulaiman R.S. (1998). Effects of mercury on wildlife: A comprehensive review. Environ. Toxicol. Chem..

[41] Custer T.W., Custer C.M., Hines R.K., Gutreuter S., Stromborg K.L. (1999). Organochlorine contaminants and reproductive success of double-crested cormorants from Green Bay, Wisconsin, USA. Environ. Toxicol. Chem..

[42] Burger J., Gochfeld M. (2000). Metals in albatross feathers from midway atoll: Influence of species, age, and nest location. Environ. Res..

[43] Bouton S.N., Frederick P.C., Spalding M.G., McGill H. (1999). Effects of chronic low concentrations of dietary methylmercury on the behavior of juvenile Great Egrets. Environ. Toxicol. Chem..

[44] Eisler R. (1987). Mercury Hazards to Fish, Wildlife and Invertebrates: A Synoptic Review.

[45] Elliott J.E., Scheuhammer A.M., Leighton F.A., Pearce P.A. (1992). Heavy metal and metallothionein concentrations in Atlantic Canadian seabirds. Arch. Environ. Contam. Toxicol..

[46] Spalding M.G., Frederick P.C., McGill H.C., Bouton S.N., McDowell L.R. (2000). Methylmercury accumulation in tissues and its effects on growth and appetite in captive great egrets. J. Wildl. Dis..

[47] Burger J., Gochfeld M. (2000). Effects of lead on birds (Laridae): A review of laboratory and field studies. J. Toxicol. Environ. Health.

[48] Wiener J.G., Spry D.J., Beyer W.N., Heinz G.H., Redmon-Norwood A.W. (1996). Toxicological significance of mercury in freshwater fish. Environmental Contaminants in Wildlife: Interpreting Tissue Concentrations.

[49] Eisler R. (1985). Cadmium Hazards to Fish, Wildlife and Invertebrates: A Synoptic Review.

[50] Jackson A.K., Evers D.C., Etterson M.A., Condon A.M., Folsom S.B., Detweiler J., Schmerfeld J., Cristol D.A. (2011). Mercury exposure affects the reproductive success of a free-living terrestrial songbird, the Carolina Wren (*Thryothorus ludovicianus*). The Auk.

[51] Heinz G.H., Beyer W.M., Heinz W.M. (1996). Selenium in birds. Environmental Contaminants in Wildlife: Interpreting Tissue Concentrations.

[52] Ackerman J.T., Eagles-Smith C.A. (2009). Selenium bioaccumulation and body condition in shorebirds and terns breeding in San Francisco Bay, California, USA. Environ. Toxicol. Chem..

